# Puerarin Relieves Paclitaxel-Induced Neuropathic Pain: The Role of Na_v_1.8 β1 Subunit of Sensory Neurons

**DOI:** 10.3389/fphar.2018.01510

**Published:** 2019-01-07

**Authors:** Xiao-Long Zhang, Xian-Ying Cao, Ren-Chun Lai, Man-Xiu Xie, Wei-An Zeng

**Affiliations:** ^1^State Key Laboratory of Oncology in South China, Collaborative Innovation Center for Cancer Medicine, Department of Anesthesiology, Sun Yat-sen University Cancer Center, Guangzhou, China; ^2^College of Food Science and Technology, Hainan University, Haikou, China; ^3^State Key Laboratory of Marine Resources Utilization in South China Sea, Hainan University, Haikou, China

**Keywords:** puerarin, paclitaxel, dorsal root ganglion, neuropathic pain, Na_v_1.8, β1 subunit

## Abstract

Currently there is no effective treatment available for clinical patients suffering from neuropathic pain induced by chemotherapy paclitaxel. Puerarin is a major isoflavonoid extracted from the Chinese medical herb kudzu root, which has been used for treatment of cardiovascular disorders and brain injury. Here, we found that puerarin dose-dependently alleviated paclitaxel-induced neuropathic pain. At the same time, puerarin preferentially reduced the excitability and blocked the voltage-gated sodium (Na_v_) channels of dorsal root ganglion (DRG) neurons from paclitaxel-induced neuropathic pain rats. Furthermore, puerarin was a more potent blocker of tetrodotoxin-resistant (TTX-R) Na_v_ channels than of tetrodotoxin-sensitive (TTX-S) Na_v_ channels in chronic pain rats’ DRG neurons. In addition, puerarin had a stronger blocking effect on Na_v_1.8 channels in DRG neurons of neuropathic pain rats and β1 subunit siRNA can abolish this selective blocking effect on Na_v_1.8. Together, these results suggested that puerarin may preferentially block β1 subunit of Na_v_1.8 in sensory neurons contributed to its anti-paclitaxel induced neuropathic pain effect.

## Introduction

Paclitaxel, a microtubule-targeted agent extracted from *Taxus brevifolia* (Wani et al., 1971), is commonly used to treat various types of cancers, such as non-small cell lung, ovarian, and breast cancers (Armstrong et al., 2006; Sandler et al., 2006; Miller et al., 2007). Although the mechanisms are not well understood, the use of paclitaxel is often related with peripheral neuropathy that mainly manifests as tingling, numbness, cold, and burning/shooting pain (Dougherty et al., 2004; Loprinzi et al., 2011; Reeves et al., 2012). A previous study showed that application of paclitaxel could enhance excitability of primary sensory neurons in dorsal root ganglion (DRG) (Zhang and Dougherty, 2014). The accumulation of paclitaxel in the peripheral nervous system may damage the peripheral fibers (Cavaletti et al., 2000; Dougherty et al., 2004), the terminals of neurons that mediate the signals of pain stimuli and cause neuropathic pain which seriously affects the patients’ quality of life and the process of chemotherapy (Dougherty et al., 2004). There is currently no effective therapeutic drug to avoid or minimize the neuropathic pain induced by paclitaxel.

Voltage-gated sodium (Na_v_) channels are necessary for the generation of action potentials (APs), which are critical for the transduction of nociceptive signals. Among the nine pore-forming α subunits of Na_v_ channels (Catterall et al., 2005), Na_v_1.6, Na_v_1.7, Na_v_1.8, and Na_v_1.9 are present in DRG neurons and contribute to somatosensory signal transmission (Dib-Hajj et al., 2010). Particularly, the tetrodotoxin-resistant (TTX-R) Na_v_1.8 is critical to pain transmission (Lai et al., 2004). A study on Na_v_1.8 gene knockout mice demonstrated the requirement for Na_v_1.8 in nociception (Akopian et al., 1996). Furthermore, knock down of Na_v_1.8 impairs nerve injury- and complete Freund’s adjuvant (CFA)-induced chronic pain (Porreca et al., 1999; Yu et al., 2011). Deletion of Na_v_1.8 in DRG neurons inhibits capsaicin, mustard oil and nerve growth factor (NGF)-induced thermal hyperalgesia (Kerr et al., 2001; Laird et al., 2002). Auxiliary β subunits, which are expressed in DRG neurons (Chahine et al., 2005), interact with the α-subunit to mediate the gating properties and current densities of Na_v_ channels. Knockout of the β2 subunit decreases TTX-S currents but not TTX-R currents in DRG neurons (Lopez-Santiago et al., 2006). Additionally, Co-expression of the β1 subunit increased Na_v_1.8 current density in HEK293 cells (Zhao et al., 2011).

Puerarin is a major isoflavonoid extracted from the Chinese medical herb kudzu root, which has been used for the treatment of cardiovascular disorders (Yeung et al., 2006; Gao et al., 2007) and brain injury in China (Gao et al., 2009). Clinical studies have shown that puerarin could ameliorate prostatodynia (YunLong, 2008) and burn-related procedural pain (Li et al., 2011; Zhang et al., 2013) in patients. Experimental studies demonstrate that puerarin attenuates nerve injury-induced neuropathic pain by modulating P2X3 receptors (Xu et al., 2012) or activating ERK and CREB pathways (Zhao et al., 2016). Whether puerarin could treat the neuropathic pain caused by chemotherapy paclitaxel has not been investigated. Here, we revealed that puerarin may preferentially inhibit the β1 subunit of Na_v_1.8 and reduce the excitability of DRG neurons contributes to its anti-paclitaxel induced neuropathic pain effect. Our findings may provide a potential mechanism by which puerarin inhibits neuropathic pain induced by paclitaxel.

## Materials and Methods

### Subjects

Male Sprague-Dawley rats (100–250 g) were purchased from the Institute of Experimental Animals of Sun Yat-sen University. The rats were individually housed in separate cages in a temperature-controlled (24 ± 1°C) and humidity-controlled (50–60%) room, under a 12/12-h light/dark cycle and with *ad libitum* access to sterile water and standard laboratory chow. All animal experimental procedures were approved by the local Animal Care Committee and were carried out in accordance with the guidelines of the National Institutes of Health on animal care and the ethical guidelines (Zimmermann, 1983). All animals were randomly assigned to different experimental or control conditions.

### Drug Administration

One milliliter of (6 mg) paclitaxel (taxol, 30 mg in 5 ml, Bristol-Myers Squibb, New York, NY, United States) was diluted with 2 ml sterile saline and injected intraperitoneally (*i.p.*) at a dosage of 8 mg/kg on three alternate days (days 1, 4, and 7). Control animals received an equivalent volume of sterile saline.

The chronic indwelling peri-DRGs catheter systems were implanted according to the method described previously (Lyu et al., 2000). After animals were anesthetized under halothane (2%), Peri-DRGs catheters filled with saline were constructed from sterile gelfoam aseptically cut into 20-mm (L) × 7-mm (W) × 6-mm (H) strips. One end was bisected (3.5 mm W) to a depth of 1 cm to allow a 5-cm sterile polyethylene (PE-10) tube to be sutured inside. The assembly was inserted around the L4-6 DRGs. Vehicle or puerarin at different concentrations (0.1, 1.0, and 10 μM in 15 μl volume) was injected slowly (2 min) through the PE-10 tube. Special care was paid to prevent infection.

Intrathecal injection of Na_v_β1 siRNA (50 μg/15 μl, Ribobio, China) or scramble siRNA (50 μg/15 μl, Ribobio, China) was performed 30 min prior to paclitaxel administration for 10 consecutive days. Intrathecal injection was performed as previously described (Xu et al., 2007). Briefly, halothane (2%) was used for anesthesia. After the dura was probed with an 8G needle, a polyethylene-10 (PE-10) catheter was inserted into the subarachnoid space of the rat through the L5/L6 intervertebral space, and the tip of the catheter was placed at the L5 spinal segmental level. After intrathecal implantation, the rats were allowed to recover from surgery for at least 5 days prior to subsequent drug injection. Any rats exhibiting hind limb paralysis or paresis after surgery were excluded from the study.

### Behavioral Tests

#### Mechanical Allodynia

Each rat was loosely restrained in a plastic box on a metal mesh and allowed to acclimate for at least 15 min per day for three consecutive days. Mechanical sensitivity was assessed using von Frey hairs with the up-down method as described previously (Chaplan et al., 1994). In brief, each rat was placed in a transparent Plexiglas testing chamber positioned on a wire mesh floor and allowed 20 min for habituation. Each stimulus consisted of a 6–8 s application of the von Frey hair to the lateral surface of the paw of paclitaxel rats with a 5 min interval between stimuli. Brisk withdrawal or licking of the paw in response to the stimulus was considered a positive response. The operator who performed the behavioral tests was blinded to all treatments.

#### Thermal Hyperalgesia

Thermal hypersensitivity was measured using a plantar test (7370, UgoBasile, Comeria, Italy) according to the method described by Hargreaves et al. (1988). Briefly, a radiant heat source beneath a glass floor was aimed at the fat part of the heel on the plantar surface of the hind paw. Four measurements of latency were taken for each hind paw with a minimal value of 0.5 s and a maximum of 25 s in each test session. Each hind paw was tested alternately with at least 5 min intervals between consecutive tests. The four measurements of latency per side were averaged as the results per test.

### DRG Neuron Preparation

Dorsal root ganglion neurons were dissociated via enzyme digestion as previously described with slight modifications (Xie et al., 2017; Zhang et al., 2018). In brief, L4-6 DRGs in control and paclitaxel rats were freed from their connective tissue sheaths and broken into pieces with a pair of sclerotic scissors in DMEM/F12 medium (Gibco, United States) under low temperature (in a mixture of ice and water). DRG neurons were plated on glass cover slips coated with poly-L-lysine (Sigma, United States) in a humidified atmosphere (5% CO_2_, 37°C) following enzymatic and mechanical dissociation. The cells were used for electrophysiological recordings approximately 4 h to 24 h after plating.

### Electrophysiological Recordings

Whole-cell patch clamp recordings were performed with an EPC-10 amplifier and the PULSE program (HEKA Electronics, Lambrecht, Germany) as previously described (Xie et al., 2017). Currents were recorded with glass pipettes (1–3 MΩ resistance) fabricated from borosilicate glass capillaries using a Sutter P-97 puller (Sutter Instruments, Novato, CA, United States). Membrane currents were filtered at 10 kHz and sampled at 50 kHz. Voltage errors were minimized by using 80–90% series resistance compensation. The Na^+^ currents were recorded in DRG neurons. The neurons with a leak current of >500 pA or a series resistance of >10 MΩ were excluded. We recorded the excitability of DRG neurons (20–35 μm in diameter), which are proved to express both TTX-S and TTX-R Na_v_ channels (Scholz et al., 1998; Fang et al., 2002; Djouhri et al., 2003). TTX-R Na^+^ currents were recorded in neurons (diameter < 25 μm) which TTX-R Na^+^ currents were prevailed (Scholz et al., 1998). TTX-S Na^+^ currents were recorded in neurons (diameter > 40 μm) which were mainly displayed TTX-S Na^+^ currents (Scholz et al., 1998). As the DRG neurons <25 μm are cell bodies of C-fibers and those >35 μm are low threshold A-fibers (Chahine and O’Leary, 2014). It has been well established that in physiological conditions, C-fibers and Aδ-fibers conduct pain signals, while Aβ-fibers convey low threshold touch and vibration sense. In neuropathic pain condition, however, the activation of Aβ-fibers is also able to drive spinal pain signal pathways (Devor, 2009). For voltage clamp experiments on DRG neurons, the extracellular solution contained (in mM): 30 NaCl, 20 TEA-Cl, 90 choline chloride, 3 KCl, 1 CaCl_2_, 1 MgCl_2_, 10 HEPES, 10 glucose, and 0.1 CdCl_2_ (adjusted to pH 7.3 with Tris base). The pipette solution contained (in mM): 135 CsF, 10 NaCl, 10 HEPES, 5 EGTA, and 2 Na_2_ATP (adjusted to pH 7.2 with CsOH). For current clamp experiments on DRG neurons, the bath solution contained (in mM): 140 NaCl, 3.5 KCl, 1 MgCl_2_, 2 CaCl_2_, 10 Glucose, 10 HEPES, 1.25 NaH_2_PO_4_, pH adjusted to 7.4 with NaOH and the pipette solution contained (in mM): 40 NaCl, 140 K-Cluconate, 0.5 CaCl_2_, 10 HEPES, 5 EGTA, pH adjusted to 7.2 with KOH. The osmolality of all solutions was adjusted to 310 mOsm. We included 300 nM TTX to block TTX-sensitive currents for Na_v_1.8 recording.

### Pulse Protocols and Current Measures

The APs of DRG neurons were recorded in current clamp mode. APs were elicited by a series of depolarizing currents from 0 to 700 pA (150 ms) in 50 pA step increments to measure the current threshold (rheobase). The current that induced the first AP was defined as 1× rheobase. To test the effects of puerarin on firing rates, the mean number of APs elicited by a depolarizing current (1 s duration) at double-strength of rheobase (2 × rheobase) was measured before and 15 min after puerarin (5 μM) application. After the whole-cell recording was established, the membrane potential was held at -90 mV, then a Na^+^ current was elicited at a -10-mV depolarization potential from DRG neurons. For the calculation of I–V curves, the voltage-dependence of Na_v_ channel activation was evoked from a holding potential of -90 mV and then depolarized from -120 mV to +100 mV at 5-mV steps. Steady-state inhibition of Na^+^ currents was measured using a double pulse pattern consisting of a series of two depolarizing test pulses to -10 mV for 20 ms. From a holding potential of -120 mV, the first depolarizing test pulse was followed by a hyperpolarizing conditioning inter pulse to half inactivation voltage (10 s interval), followed by a 20 ms recovery period at -120 mV and then a second depolarizing test pulse to -10 mV for 20 ms. The pulse pattern was repeated at 30 s intervals and the peak amplitude of the inward current at both test pulses was measured. To determine the condition voltage at which approximately 50% were inactivated, Na^+^ current was measured using a step pulse protocol from -120 mV to -40 mV stepped by 10 mV over a period of 8 s, and then was recorded at a voltage of 0 mV. This was repeated at 20 s intervals from a holding potential of –120 mV. The peak amplitude of the inward current was measured. To test the use-dependent blockage of Na_v_ channels by puerarin, the holding potential was -90 mV to the test pulse of -10 mV at different frequencies (1, 3, and 10 Hz). The amplitude of currents evoked by the *n*th impulse was normalized to the current evoked by the first impulse. For building activation curves, the cell was clamped at a holding potential of -90 mV and a prepulse voltage to -120 mV for 200 ms was applied, and Na^+^ current was elicited by a stepped depolarization test voltage pulse from -80 mV to 100 mV for 50 ms. To build steady state fast inactivation curves, the cell was clamped at a holding potential of -90 mV, a stepped prepulse from -120 mV to 40 mV with 5 mV increments for 1,000 ms was applied, and the Na^+^ current was recorded at a voltage of 0 mV. For building steady state slow inactivation, DRG neurons in the whole-cell configuration were held at -80 mV, stepped to pre-pulse potentials between -120 mV and +50 mV (10 mV increments, 5 s duration) and hyperpolarized to -80 mV for 1 s to convert fast-inactivated channels in the resting state. Time constants for recovery from the inactivation of Na_v_ channels was measured with a double-pulse protocol. A first pulse (P1) for 250 ms to -10 mV caused inactivation, and Na^+^ current evoked by the test pulse (P2) to -10 mV after variable intervals was compared with *I*_Na,P1_ of the same episode. Nav1.7 current was isolated from total Na^+^ currents by subtraction of the ProTx II-resistant Na^+^ currents from total current using a previously published subtraction protocol (Schmalhofer et al., 2008; Li et al., 2018). Neurons were held at -90 mV, and activation was evoked with a 15 ms step to potentials ranging from -90 mV through +45 mV in 5 mV increments before and after ProTx II administration. Current density was calculated by normalizing maximal peak currents with cell capacitance.

To obtain half maximal inhibitory concentration (IC_50_) values, the fractional blocks obtained at different drug concentrations were fitted with the Hill equation: *E* = *E*_max_/ [1 + (IC_50_/*C*)^b^], where *E* is the inhibition of currents in percentage at concentration *C, E*_max_ is the maximum inhibition, IC_50_ is the concentration for 50% inhibition of the maximum effect, and *b* is the Hill coefficient. The activation or inactivation conductance variables of *I*_Na_ were determined with normalized currents. Current activation and inactivation were fitted by the Boltzmann distribution: *y* = 1/{1 + exp [(V_m_ - V_0:5_)/*S*], where *V*_m_ is the membrane potential, *V*_0.5_ is the activation or inactivation voltage midpoint, and *S* is the slope factor. The relation of 1/τ_block_ against the concentration is described by the linear function: 1/τ_block_ = *k* [*D*] + *l*, where 1/τ_block_ is the time constant of development of the block, and *k* and *l* are the apparent rate constants for association and dissociation of the drug.

### Solutions and Chemicals

Puerarin powder (Macklin, China) was dissolved as a stock solution of 1 mM or 10 mM in distilled water and diluted with extracellular solution or sterile saline solution to different working concentrations. Puerarin solution was adjusted to pH 7.35–7.40. Tetrodotoxin (Shanghai Absin, China) was dissolved as a stock of 1 mM in acetic acid aqueous solution and diluted to a working concentration of 300 nM for recordings on Na_v_1.8 current. A-803467 (Alomone Labs, Israel) was dissolved as a stock of 10 mM in DMSO and diluted to 1 μM with extracellular solution.

### Western Blot

Rats were deeply anesthetized with sodium pentobarbital (50 mg/kg, *i.p.*) at various time points. The L4-6 DRGs from rats were dissected and homogenized in cold RIPA buffer [50 mM Tris-HCl (pH 7.4), 150 mM NaCl, 0.1% Triton X-100, 1% sodium deoxycholate, 0.1% SDS, 10 mM NaF, 1 mM EDTA, 1 mM PMSF, and 1 mg/ml leupeptin]. The protein samples were separated via gel electrophoresis (SDS-PAGE) and transferred onto a PVDF membrane. The membranes were placed in blocking buffer for 1 h at room temperature and incubated in a primary antibody against Na_v_β1 (1:400, Alomone Labs, Israel), Na_v_1.8 (1:200, Alomone Labs, Israel), or β-actin (1:2000, CST, United States) overnight at 4°C. Next, the membranes were incubated in HRP-conjugated secondary antibody. An enhanced chemiluminescence (ECL) solution (Pierce, United States) was used to detect the immunocomplexes. Each band was quantified with a computer-assisted imaging analysis system (NIH ImageJ).

### Immunohistochemistry and Structured Illumination Microscopy

Rats were perfused with 4% paraformaldehyde (PFA). The L4-6 DRGs were dissected and post-fixed in 4% PFA for 1 h. Then the tissues were dehydrated in 30% sucrose 3 days and embedded for cryostat sectioning. The cryostat sections (12 μm) were blocked with 3% donkey serum in 0.3% Triton X-100 for 1 h at room temperature and incubated in primary antibodies against Na_v_β1 (1:200, rabbit, Alomone Labs, Israel), Na_v_1.8 (1:200, mouse, Abcam, United Kingdom) at 4°C overnight. After the primary antibody incubation, tissue sections were washed three times in 0.01 M PBS and then incubated in Cy3-conjugated (1:400, donkey anti-mouse, Jackson ImmunoResearch, United States) and FITC-conjugated (1:200, donkey anti-rabbit, Jackson ImmunoResearch, United States) secondary antibodies for 1 h at room temperature. The sections were rinsed with 0.01 M PBS three times and mounted on a coated slide, and air-dried. Three-dimensional super-resolution images were captured using a three-dimensional structured illumination microscope with the N-SIM System with an oil immersion objective lens CFI SR (Apochromat TIRF × 100, 1.49 numerical aperture, Nikon, Japan) and images were post-processed with Nikon NIS-Elements software.

### RNA Extraction and Real-Time Quantitative PCR

Total RNA was extracted from the rat L4-6 DRGs with Trizol reagent (Invitrogen, United States). Reverse transcription was performed with oligo-dT primers and M-MLV reverse transcriptase (Promega, United States) according to the manufacturer’s protocol. Primer sequences of the Na_v_β1 mRNA and β-actin for PCR reactions are listed in Table 1. Real-time quantitative PCR was performed with SYBR Green qPCR SuperMix (Invitrogen, United States) and an ABI PRISM7500 Sequence Detection System. The reactions were set up based on the manufacturer’s protocol. PCR reactions conditions were incubation at 95°C for 3 min followed by 40 cycles of thermal cycling (10 s at 95°C, 20 s at 58°C, and 10 s at 72°C). The relative expression ratio of mRNA was quantified via the 2^-ΔΔCT^ method.

**Table 1:** Specific primer sequences.

Gene	Primer	Sequence
Na_v_β1	Forward	5′-TCTACCGCCTGCTCTTCTTC-3′
	Reverse	5′-GGCAGCGATCTTCTTGTAGC-3′
β-Actin	Forward	5′-AGGGAAATCGTGCGTGACAT-3′
	Reverse	5′-GAACCGCTCATTGCCGATAG-3′

### siRNA Preparation and Screening

Three siRNA duplexes targeting the rat Na_v_β1 gene were designed with the siRNA Target Finder and DesignTool and were commercially obtained from Ribobio (China). The sequences of these siRNAs were as follows: siRNA1, target sequence 1:

GAAGGGCACAGAGGAATTT
5′-GAAGGGCACAGAGGAAUUUdTdT-3′ (sense)
3′-dTdTCUUCCCGUGUCUCCUUAAA-5′ (antisense)
siRNA2, target sequence 2: GCCAACAGAGATATGGCAT
5′-GCCAACAGAGAUAUGGCAUdTdT-3′ (sense)
3′-dTdTCGGUUGUCUCUAUACCGUA-5′ (antisense)
siRNA3, target sequence 3: CCATTACTTCCGAGAGCAA
5′-CCAUUACUUCCGAGAGCAAdTdT-3′ (sense)
3′-dTdTGGUAAUGAAGGCUCUCGUU-5′ (antisense).

A siRNA with no homology to the Na_v_β1 gene was used as a control (Scramble). The DRG neurons were transfected with siRNA using lipofectamine 2000 (Invitrogen, United States) according to the manufacturer’s instructions. Na_v_β1 expression levels were determined using qPCR. Compared with the blank control, the cells treated with siRNA1, siRNA2 or siRNA3 exhibited suppression of Na_v_β1 messenger RNA (mRNA) expression by 85.1 ± 5.3%, 59.9 ± 4.7%, and 42.7 ± 2.1%, respectively, when measured 24 h after transfection. Hence, the synthesized Na_v_β1 siRNA1 was chosen for subsequent experiments.

### Co-immunoprecipitation

The dissected tissues were lysed in cold RIPA buffer [20 mM Tris-HCl (pH 7.5), 150 mM NaCl, 0.1% Triton X-100, 1% sodium deoxycholate, 10 mM NaF, 1 mM EDTA, 1 mM PMSF, and 1 mg/ml leupeptin]. The lysate was centrifuged and take 5% of the supernatant for input sample. The remaining supernatant was precipitated with 1–5 μg Na_v_1.8 or Na_v_β1 antibody at 4°C overnight and afterward protein A/G beads (GE Healthcare) at 4°C for 4 h. The immunoprecipitated sample was denatured and prepared for immunoblotting. The immunoprecipitation was performed with antibodies against Na_v_β1 or Na_v_1.8 antibody.

### Statistical Analysis

All data were expressed as the mean ± SD. Mathematical curve fitting and statistical analyses were performed with Prism 6 (GraphPad Software Inc., San Diego, CA, United States) and SPSS 13.0 (SPSS, Chicago, IL, United States). For Western blot, electrophysiological and behavioral data were analyzed by two-tailed, independent Student’s *t*-test, two-tailed paired Student *t*-test, one-way ANOVA followed by Tukey *post hoc* test and two-way repeated measures ANOVA followed by Bonferroni *post hoc test* was carried out. Threshold for statistical significance was *P* < 0.05. Although no power analysis was performed, the sample size was determined according to previous publications in pain-associated behavior and molecular studies.

## Results

### Puerarin Attenuates Mechanical Allodynia and Thermal Hyperalgesia Induced by Paclitaxel

**FIGURE 1:**
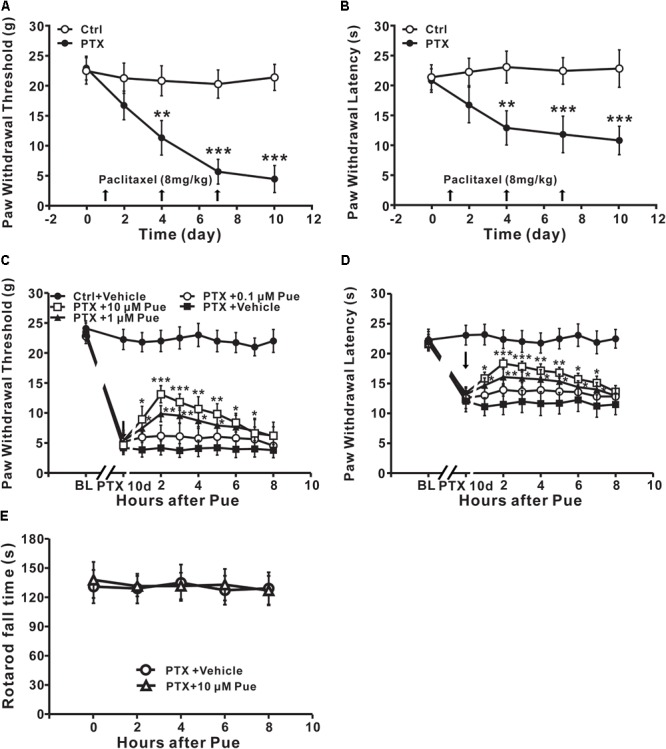
Local application of puerarin attenuates mechanical allodynia and thermal hyperalgesia induced by paclitaxel (Ctrl, control; PTX, paclitaxel; Pue, puerarin). **(A,B)** Paclitaxel caused significant mechanical allodynia **(A)** and thermal hyperalgesia **(B)**. *n* = 12 rats in each group. ^∗∗^*P* < 0.01, ^∗∗∗^*P* < 0.001 compared with the control group. **(C)** Puerarin dose-dependently relieved paclitaxel-induced mechanical allodynia (day 10). *n* = 18 rats in each group. ^∗^*P* < 0.05, ^∗∗^*P* < 0.01, ^∗∗∗^*P* < 0.001 compared with the paclitaxel + vehicle group (BL: baseline). **(D)** Puerarin dose-dependently relieved paclitaxel-induced thermal hyperalgesia (day 10). *n* = 12 rats in each group. ^∗^*P* < 0.05, ^∗∗^*P* < 0.01, ^∗∗∗^*P* < 0.001 compared with the paclitaxel + vehicle group (BL, baseline). **(E)** Motor performance was not changed after the local application of 10 μM puerarin. *n* = 10 rats in each group.

First, we investigated whether application of puerarin onto L4-6 DRGs could relieve neuropathic pain induced by paclitaxel. Consistent with our previous study (Li et al., 2015), abdominal injection paclitaxel (3 × 8 mg/kg, cumulative dose 24 mg/kg) induced significant mechanical allodynia (Figure 1A) and thermal hyperalgesia (Figure 1B). A single local application of puerarin dose-dependently attenuated mechanical allodynia (Figure 1C) and thermal hyperalgesia (Figure 1D) induced by paclitaxel. Puerarin at 1 μM or 10 μM was effective on neuropathic pain. To test whether puerarin affected pain behaviors by obstructing motor function, we performed a rotarod experiment before and after local application of puerarin at the highest dose. The results showed that 10 μm puerarin did not affect motor coordination (Figure 1E).

### Puerarin Reduces the Hypersensitivity of DRG Neurons Induced by Paclitaxel

**FIGURE 2:**
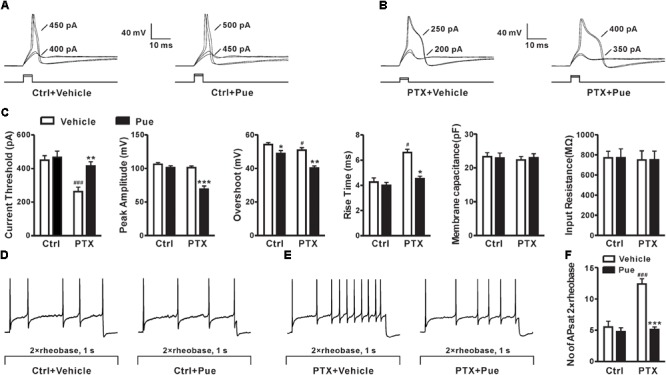
Puerarin reduces paclitaxel-induced hypersensitivity in DRG neurons (Ctrl, control; PTX, paclitaxel; Pue, puerarin). **(A,B)** The representative traces show the thresholds of action potentials evoked by current injections to DRG neurons from control rats **(A)** and paclitaxel rats (day 10) **(B)** before and 15 min after the application of 5 μM puerarin. **(C)** Comparisons of the threshold of action potentials, the peak amplitude, the overshoot, the rise time, the membrane capacitance and the input resistance before and after puerarin treatment. **(D,E)** The typical traces indicate the action potentials elicited by 2 × rheobase for 1 s in DRG neurons of control rats **(D)** and paclitaxel rats (day 10) **(E)** before and after puerarin. **(F)** The summary data show the comparison of the numbers of action potentials in control and paclitaxel (day 10) rats with or without puerarin. *n* = 12 neurons in each group. ^∗^*P* < 0.05, ^∗∗^*P* < 0.01, ^∗∗∗^*P* < 0.001 compared to the corresponding vehicle groups; ^#^*P* < 0.05, ^###^*P* < 0.001 compared corresponding to control groups.

As we known that the increased excitability of DRG neurons play a critical role in the development of neuropathic pain (Ratte and Prescott, 2016). Whether puerarin alleviates paclitaxel-induced neuropathic pain by affecting the excitability of DRG neurons remains unclear. Due to 1–10 μM puerarin significantly remitted paclitaxel-induced neuropathic pain, we then test the effect of 5 μM puerarin on the excitability of DRG neurons (20–35 μm in diameter), which are proved to express both TTX-S and TTX-R Na_v_ channels (Scholz et al., 1998; Fang et al., 2002; Djouhri et al., 2003). The APs were elicited by a series of depolarizing currents from 0 to 700 pA (150 ms) in 50 pA step increments under current clamp mode. The minimal current that evoked an AP was defined as threshold (rheobase) (Figures 2A,B). We found that the half-width was increased and the threshold was decreased after paclitaxel treatment (Figures 2B,C). Importantly, puerarin significantly prevented paclitaxel-induced threshold reduction and increased the half-width of AP in DRG neurons, while the change was not detected in controls (Figures 2B,C). Puerarin reduced the peak amplitude and the rise time in the paclitaxel group but not in the control group (Figure 2C). Puerarin decreased overshoot in both control and paclitaxel groups but did not affect the membrane capacitance or the input resistance in the two groups (Figure 2C). To test the effects of puerarin on the firing rates of DRG neurons, the number of APs elicited by a depolarizing current (1 s duration) at double-strength of rheobase (2 × rheobase) was measured before and after puerarin exposure. The result showed that paclitaxel increased the number of APs in DRG neurons (Figures 2D–F). And puerarin markedly reversed the increased number of APs in DRG neurons induced by paclitaxel, whereas no difference was detected in control neurons (Figures 2D–F). Thus, the inhibitory effect of puerarin on the over-excitatory of DRG neurons may contribute to the relief of neuropathic pain.

### Puerarin Preferentially Blocks Resting Na_v_ Channels of DRG Neurons in Paclitaxel Rats

**FIGURE 3:**
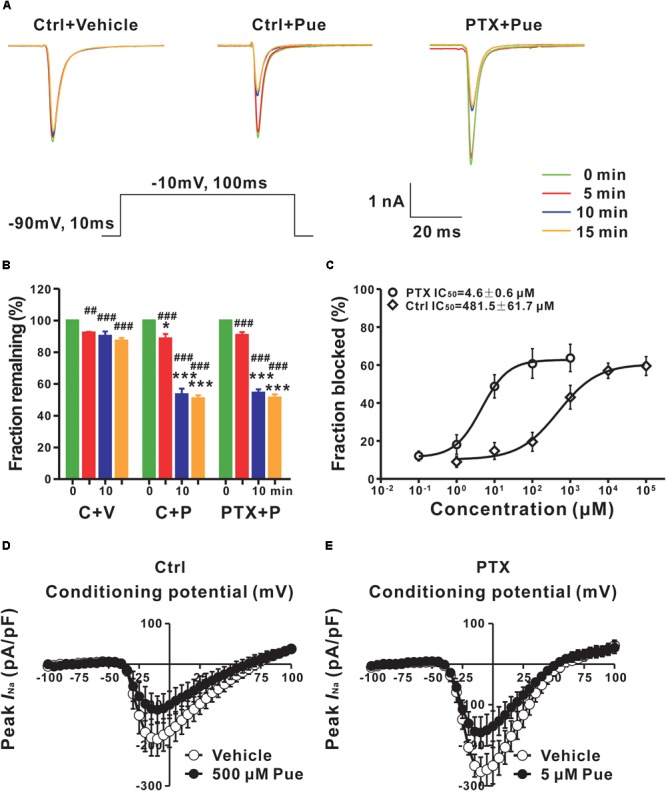
Puerarin preferentially blocks resting Na_v_ channels in DRG neurons of paclitaxel (day 10) rats (Ctrl, control; PTX, paclitaxel; Pue, puerarin). **(A)** The currents were recorded before and 5, 10, 15 min after the external application of vehicle or puerarin at corresponding IC_50_ concentrations. Currents traces evoked from a holding potential of –90 mV to the test pulse of –10 mV (insert). **(B)** The summary data show the inhibitory rates of puerarin in indicated groups and time points after puerarin application (C, control; P, puerarin; V, vehicle; *n* = 8 neurons in each group). ^∗^*P* < 0.05, ^∗∗∗^*P* < 0.001 compared with corresponding C+V groups; ^##^*P* < 0.01, ^###^*P* < 0.001 compared with corresponding 0 min. **(C)** IC_50_ values of puerarin on resting Na_v_ channels of DRG neurons in control (*n* = 8 neurons in each data point) and paclitaxel (*n* = 8 neurons in each data point) groups. **(D,E)** The currents were recorded before and 15 min after puerarin application at corresponding IC_50_ concentrations in control **(D)** and paclitaxel **(E)** groups. *n* = 9 neurons in each group.

The Na_v_ channel is the basis for the generation and conduction of APs; therefore, we investigated the effect of puerarin on Na_v_ channels. It is well-known that Na_v_ channels have three types of states: resting, open and inactivated. Na_v_ channels rapidly transform from the resting state to the open, inactivated state and eventually back to the resting state (Aldrich et al., 1983). Blocking the different states or the transformation between two states may lead to diverse pharmacological effects (Hille, 1977). To determine the effect of puerarin on Na_v_ channels, we first examined the effect of puerarin on resting Na_v_ channels. We recorded the currents before and 5, 10, 15 min after the external application of vehicle or puerarin at different concentrations. The results demonstrated that *I*_Na,peak_ decreased significantly with time after puerarin treatment in each group. Compared with vehicle, the decrease in *I*_Na,peak_ reached a plateau 10–15 min after puerarin treatment, and no difference was detected between them (Figures 3A,B). To obtain the IC_50_ of puerarin on resting Na_v_ channels, the net inhibitory rate (subtracting the reduction in *I*_Na,peak_ in vehicle-treated neurons from that in puerarin treated neurons) was recorded 15 min after puerarin treatment. The IC_50_ of puerarin for resting Na_v_ channels was 481.5 ± 61.7 μM in control rats and 4.6 ± 0.6 μM in paclitaxel rats (Figure 3C). Namely, the potency of puerarin blockage on resting Na_v_ channels in paclitaxel group was 105 times higher than that in control group (Table 2), suggesting that puerarin preferentially blocked resting Na_v_ channels in DRG neurons in a neuropathic pain state. Meanwhile, the *I–V* relationship showed that *I*_Na,peak_ was reduced by approximately 50% by puerarin at the IC_50_ concentration in each group (Figures 3D,E). Additionally, the maximal inhibition rates induced by puerarin at 10 mM in the control group and at 0.1 mM in paclitaxel group were less than 72%, suggesting that the blockage of resting Na_v_ channels by puerarin was incompleted (Figure 3C).

**Table 2:** The IC_50_ values in different models of DRG neurons.

	Resting state	Inactivated state
Ctrl	481.5 ± 61.7 μM	50.8 ± 6.4 μM
PTX	4.6 ± 0.6 μM	103.9 ± 11.7 nM
TTX-S	11.8 ± 1.6 μM	352.9 ± 39.1 nM
TTX-R	1.1 ± 0.2 μM	25.8 ± 3.1 nM
Ctrl Na_v_1.8	365.1 ± 37.3 μM	30.4 ± 3.2 μM
PTX Na_v_1.8	602.4 ± 59.6 nM	13.5 ± 1.4 nM
siRNA	279.7 ± 23.3 μM	18.6 ± 1.6 μM
Scramble	521.4 ± 53.2 nM	21.2 ± 2.5 nM

### Puerarin Is More Effective on Inactivated Na_v_ Channels of DRG Neurons in Paclitaxel Rats

**FIGURE 4:**
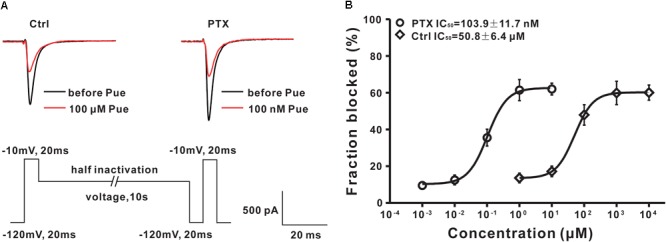
Puerarin preferentially blocks inactivated Na_v_ channels in DRG neurons of paclitaxel (day 10) rats (Ctrl, control; PTX, paclitaxel; Pue, puerarin). **(A)** The representative current traces were recorded before and 15 min after puerarin application at indicated concentrations and the protocol for the inactivation of Na_v_ channels. **(B)** IC_50_ values of puerarin on inactivated Na^+^ currents of DRG neurons in control (*n* = 9 neurons in each data point) and paclitaxel (*n* = 9 neurons in each data point) groups.

Next, we investigated the effect of puerarin on inactivated Na_v_ channels. The typical current traces were recorded before and 15 min after puerarin treatment and the protocol is shown in Figure 4A. Similar to the resting Na_v_ channels, puerarin had a much more potent effect on the neurons of neuropathic pain rats, as IC_50_ values for inactivated Na_v_ channels in paclitaxel-induced neuropathic pain rats were 489-fold lower than that in control rats (Figure 4B and Table 2). Interestingly, puerarin had a stronger effect on the inactivated state than the resting state of Na_v_ channels, as the IC_50_ of puerarin for the inactivated state in the paclitaxel model was 44-fold and in control was 9-fold lower than that at the resting state in each group (Table 2). The blockage of inactivated Na_v_ channels by puerarin was also incompleted (less than 71%), as inhibition rates of *I*_Na,peak_ reached a maximum at 1 mM in the control group and at 1 μM in the paclitaxel group (Figure 4B).

### Puerarin Has a More Powerful Use-Dependent Blockage of Na_v_ Channels on DRG Neurons in Paclitaxel Rats

**FIGURE 5:**
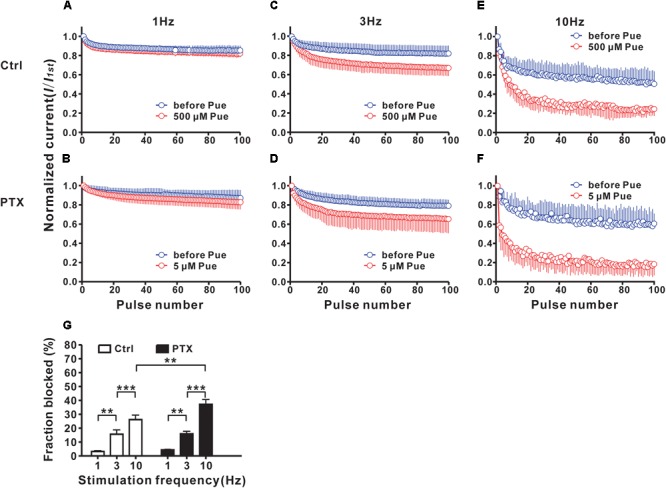
Puerarin use-dependently blocks Na_v_ channels of DRG neurons (Ctrl, control; PTX, paclitaxel; Pue, puerarin). **(A–F)** The amplitudes of *I*_Na,peak_ evoked by 100 pulses (from a holding potential of –90 mV to the test pulse of –10 mV, 40 ms) were normalized to those of the first response and plotted against pulse number. The normalized amplitudes at different frequencies in indicated groups were recorded before and 15 min after puerarin application at corresponding IC_50_ concentrations. **(G)** The comparison of the net inhibitory rates calculated with responses elicited by *100*th pulses at indicated frequencies in control and paclitaxel (day 10) groups are shown. *n* = 9 neurons in each test. ^∗∗^*P* < 0.01, ^∗∗∗^*P* < 0.001.

To study the use-dependent effects of puerarin on Na_v_ channels, Na^+^ currents evoked by 100 pulses at various frequencies were recorded before and 15 min after puerarin application in control and paclitaxel groups at corresponding IC_50_ concentrations of the resting state. No difference was detected in Na^+^ currents before and after puerarin application in all groups at 1 Hz stimulation (Figures 5A,B). When stimulated with 3 Hz or 10 Hz, the amplitudes of Na^+^ currents decreased significantly after puerarin application (Figures 5C–F). The net inhibitory rates (the ratio of peak Na^+^ currents recorded before and after puerarin application) were enhanced with increasing frequency in all groups (Figure 5G). Importantly, the net inhibitory rate in paclitaxel-induced neuropathic pain group was higher than that in control group at 10 Hz (Figure 5G). These results suggested that puerarin was more potent at blocking the high frequency Na_v_ channels that are at an open state in a neuropathic pain state.

### Puerarin Accelerates Inactivation and Delay Recovery of Na_v_ Channels in DRG Neurons

**FIGURE 6:**
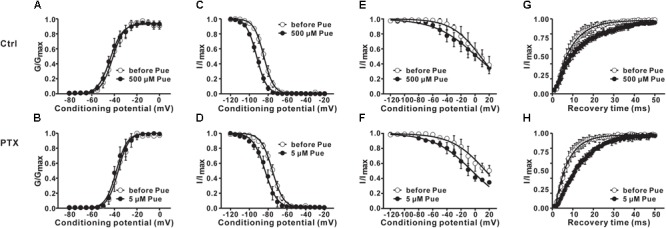
The effects of puerarin on activation, inactivation and recovery of Na_v_ channels in DRG neurons at corresponding IC_50_ concentrations (Ctrl, control; PTX, paclitaxel; Pue, puerarin). **(A,B)** Puerarin does not affect the activation curves of Na_v_ channels in control **(A)** and paclitaxel (day 10) **(B)** group (*n* = 10 neurons in each group). **(C–F)** The peak Na^+^ current (*I*_Na_) was normalized to the maximal value (*I*_Na,max_) and plotted against the conditioning pulse potential. The inactivation curves indicated that puerarin accelerated the inactivation of Na_v_ channels in two groups (*n* = 6–10 neurons in each group). **(G,H)** Puerarin slowed down the recovery from the inactivation of Na_v_ channels in each group (*n* = 10 neurons in each group). The detailed parameters of analysis are shown in Table 3.

Furthermore, we investigated the effect of puerarin on the transition between the two states of the Na_v_ channel. The changes in the activation, inactivation and recovery properties of Na_v_ channels in DRG neurons exposed to puerarin were assessed in the control and paclitaxel groups. Na^+^ currents were recorded before and 15 min after puerarin application at corresponding IC_50_ concentrations of the resting state. The data showed that puerarin had no effect on the voltage-dependency of activation (Figures 6A,B and Table 3) but induced a prominent hyperpolarizing shift of the fast (Figures 6C,D and Table 3) and slow (Figures 6E,F and Table 3) inactivation in the control and paclitaxel groups. Puerarin induced a significant delay in the recovery time from the inactivation state in two groups (Figures 6G,H and Table 3). This finding suggested that puerarin did not changed the activation, but accelerated the inactivation and delayed the recovery of Na_v_ channels in DRG neurons.

**Table 3:** Parameters for Na_v_ channels activation, inactivation, and recovery in DRG neurons.

		Activation		Fast inactivation		Slow inactivation		Recovery
		V_0.5_	k	V_0.5_	k	V_0.5_	k	τ
Ctrl	Before Pue	–41.6 ± 1.2	5.1 ± 0.5	–84.6 ± 0.3	5.3 ± 1.8	7.66 ± 0.1	20.72 ± 0.9	8.0 ± 0.5
	500μM Pue	–43.7 ± 1.4	5.5 ± 0.6	–92.2 ± 0.3^∗∗^	5.4 ± 0.1	2.12 ± 0.1^∗^	29.25 ± 1.4	12.3 ± 0.2^∗∗∗^
PTX	Before Pue	–36.6 ± 1.3	4.7 ± 0.4	–75.2 ± 0.4	6.1 ± 1.2	15.08 ± 0.2	24.33 ± 1.1	6.3 ± 0.3
	5μM Pue	–38.2 ± 1.6	4.8 ± 0.7	–81.0 ± 1.7^∗∗^	7.0 ± 0.7	–4.33 ± 0.1^∗∗^	27.22 ± 1.3	13.4 ± 0.2^∗∗∗^

*Mean values were derived from Boltzmann distribution of individual data sets as described in the Measures. ^∗^*P* < 0.05, ^∗∗^*P* < 0.01, ^∗∗∗^*P* < 0.001 vs. corresponding before Pue group.*

### Puerarin Is More Potent at Blocking Tetrodotoxin-Resistant Na_v_ Channels Than Tetrodotoxin-Sensitive Na_v_ Channels of DRG Neurons

**FIGURE 7:**
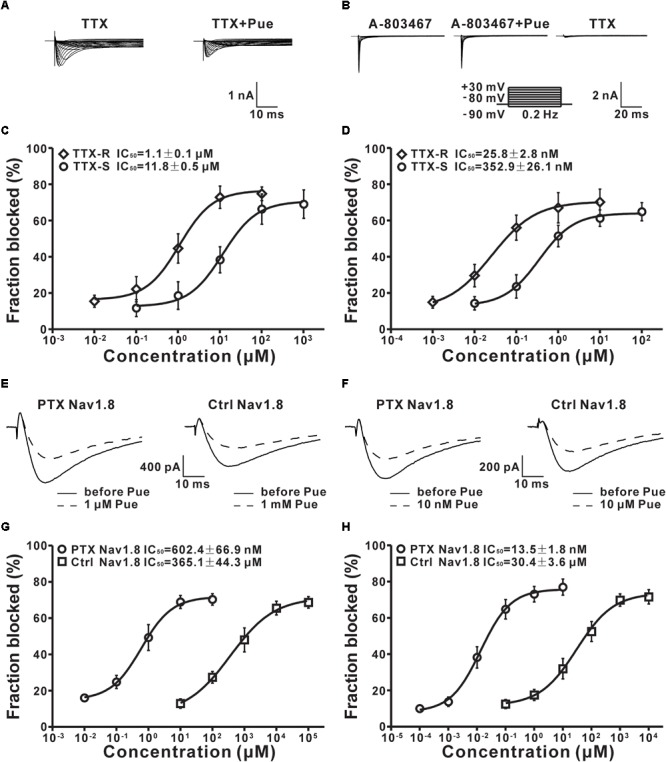
The effect of puerarin on TTX-R, TTX-S and Na_v_1.8 channels in DRG neurons (Ctrl, control; PTX, paclitaxel; Pue, puerarin). **(A)** Representative traces of TTX-R currents recorded in the presence of TTX (300 nM) or TTX (300 nM) and puerarin (0.1μM). **(B)** TTX-S currents were recorded in the presence of A-803467 (1 μM), A-803467 (1 μM) and puerarin (0.01 μM) or TTX (300 nM). **(C)** IC_50_ values of puerarin on resting TTX-R (*n* = 10 neurons in each data point) and TTX-S (*n* = 9 neurons in each data point) Na_v_ channels in DRG neurons of paclitaxel (day 10) rats. **(D)** IC_50_ values of puerarin on inactivated TTX-R (*n* = 9 neurons in each data point) and TTX-S (*n* = 9 neurons in each data point) Na_v_ channels in DRG neurons of paclitaxel (day 10) rats. **(E)** The resting Na_v_1.8 current traces were recorded before and 15 min after puerarin application at indicated concentrations. **(F)** The representative inactivated Na_v_1.8 current traces were recorded before and 15 min after puerarin application at indicated concentrations. **(G)** IC_50_ values of puerarin for resting Na_v_1.8 in control and paclitaxel (day 10) groups. *n* = 10 neurons in each data point. **(H)** IC_50_ values of puerarin for inactivated Na_v_1.8 in control and paclitaxel (day 10) groups. *n* = 10 neurons in each data point.

Na_v_ channels are divided into TTX-R and tetrodotoxin-sensitive (TTX-S) Na_v_ channels on the basis of their different sensitivities to tetrodotoxin (TTX) (Elliott and Elliott, 1993). Having shown that puerarin preferentially blocked Na_v_ channels in DRG neurons under neuropathic pain conditions, we then investigated the effect of puerarin on TTX-R and TTX-S Na_v_ channels in rats with neuropathic pain. TTX-R Na^+^ currents were recorded in neurons (diameter < 25 μm) which TTX-R Na^+^ currents were prevailed (Scholz et al., 1998) with 300 nM TTX (Figure 7A). TTX-S Na^+^ currents were recorded in neurons (diameter > 40 μm) which were mainly displayed TTX-S Na^+^ currents (Scholz et al., 1998) with A-803467(1 μM) (Anreddy et al., 2015; Tanaka et al., 2015). TTX-S Na^+^ currents were recognized originally by their relatively fast activation and inactivation kinetics and were established by adding 300 nM TTX at the end of the recordings. Only the currents reduced to 90% or more with 300 nM TTX were used for further studies (Figure 7B). The results showed that the IC_50_ for TTX-R Na_v_ channels (1.1 ± 0.2 μM) in the resting state was 11-fold lower than that for TTX-S Na_v_ channels (11.8 ± 1.6 μM) (Figure 7C and Table 2). The IC_50_ for inactivated TTX-R Na^+^ currents was 14-fold lower than that for TTX-S currents (25.8 ± 3.1 nM vs. 352.9 ± 39.1 nM) (Figure 7D and Table 2). Consistent with the total current, the IC_50_ for inactivated TTX-R and TTX-S Na^+^ currents was 43-fold and 31-fold lower than the IC_50_ for resting TTX-R and TTX-S Na^+^ currents, respectively (Table 2). The studies suggested that puerarin was more potent at blocking TTX-R than TTX-S Na_v_ channels in a neuropathic pain state, particularly in inactivated channels.

### Puerarin Has a More Potent Blocking Effect on Na_v_1.8 Subtype of DRG Neurons in Paclitaxel Rats

TTX-R Na_v_ channels include Na_v_1.8 and Na_v_1.9 subtypes in DRG neurons (Roy and Narahashi, 1992). Previous studies have suggested that Na_v_1.8 subtypes in DRG neurons are critically involved in neuropathic pain (Porreca et al., 1999; Lai et al., 2002; He et al., 2010). Therefore, we evaluated the effects of puerarin on the TTX-R Na_v_1.8 subtype in DRG neurons in paclitaxel and control rats. Na_v_1.8 currents was elicited with a test pulses were preceded by a 500 ms step to -50 mV to inactivate the Na_v_1.9 current in the presence of 300 nM TTX. The characteristic current traces recorded before and 15 min after puerarin application at indicated concentrations are shown in Figures 7E,F. The IC_50_ of puerarin for Na_v_1.8 in neuropathic pain rats induced by paclitaxel (602.4 ± 59.6 nM) was 606-fold lower than that in control rats (365.1 ± 37.3 μM) in the resting state (Figure 7G and Table 2). The IC_50_ of puerarin on Na_v_1.8 in the paclitaxel group (13.5 ± 1.4 nM) was 2252-fold lower than that in the control group (30.4 ± 3.2 μM) in the inactivated state (Figure 7H and Table 2). These findings indicated that puerarin was more effective at blocking Na_v_1.8 in a neuropathic pain state.

### Puerarin Preferentially Inhibits the Na_v_1.8 of DRG Neurons in a Neuropathic Pain State May by Blocking the β1 Subunit

**FIGURE 8:**
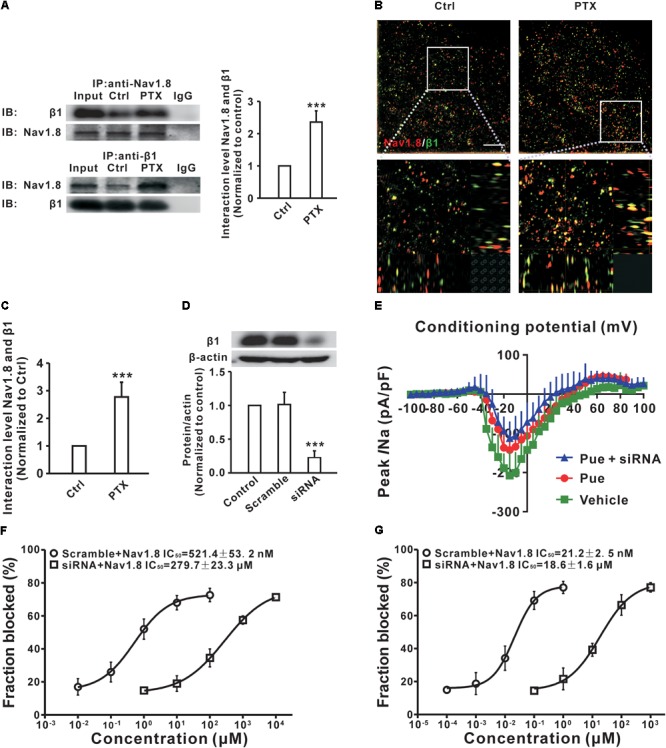
Puerarin inhibits Na_v_1.8 in a neuropathic pain state may by blocking β1 subunit in DRG neurons (Ctrl, control; PTX, paclitaxel; Pue, puerarin). **(A)** Co-IP showed that paclitaxel treatment significantly increased the binding between Na_v_1.8 and β1. *n* = 6 rats in each group. ^∗∗∗^*P* < 0.001 compared with control group. **(B,C)** High resolution image showed that the colocalization between Na_v_1.8 and β1 subunit in DRG neuron. *n* = 4 rats. ^∗∗∗^*P* < 0.001 compared with control group. Scale bars: 5 μm (top) and 1 μm (bottom). **(D)** siRNA knock down the protein expression of β1 subunit in DRG. *n* = 6 rats in each group. ^∗∗∗^*P* < 0.001 compared with scramble group. **(E)** Representative voltage-clamp recordings of Nav1.7 current in acute dissociated DRG neurons of day 10 paclitaxel-treated rats collected using a subtraction protocol (the current obtained following subtraction of the residual current following ProTx II administration from the total) the mean current density–voltage relationships are plotted in **(D)**. *n* = 10 neurons in each group. **(F,G)** The IC_50_ of puerarin on resting **(F)** or inactivated **(G)** state for Na_v_1.8 in β1 subunit knock down and scramble DRG neurons of paclitaxel (day 10) rats. *n* = 10 neurons in each data point.

It is well established that Na_v_ channels conventionally consist of a combination of pore-forming α subunit and regulatory β subunit (O’Malley and Isom, 2015) that are known as auxiliary components acting in a regulatory capacity (Isom et al., 1994). A previous study showed that the upregulation of the β1 subunit was involved in the generation of neuropathic pain (Blackburn-Munro and Fleetwood-Walker, 1999). And, co-expression of the β1 subunit increased the Na_v_1.8 current density in HEK293 cells (Zhao et al., 2011). To investigate the underlying mechanisms by which puerarin has a stronger blocking effect on Na_v_1.8 in the neuropathic pain state, we examined the interactions between Na_v_1.8 channel and β1 subunit in the DRG via co-immunoprecipitation (co-IP). In co-IP experiments with DRG protein extract, Na_v_1.8 combined with β1 subunit (Figure 8A) and the interaction between Na_v_1.8 and β1 subunit was increased in paclitaxel rats compared with control rats (Figure 8A). Further high-resolution image also confirmed that paclitaxel increased the colocalization of Na_v_1.8 and β1 subunit (Figures 8B,C). Therefore, we hypothesized that the preferentially inhibiting effect of puerarin on the Na_v_1.8 channel in the neuropathic pain state may occur via blockage of the β1 subunit. To test it, we used siRNA to knock down the β1 subunit in DRG neurons (Figure 8D) and then examined the IC_50_ values. The result showed that in paclitaxel rats the blocking effect of puerarin on Na_v_1.8 in β1 subunit knock down group (279.7 ± 23.3 μM) was 536-fold lower than in the scramble group (521.4 ± 53.2 nM) in the resting state (Figure 8F and Table 2). In the inactivated state, the blocking effect of puerarin on Na_v_1.8 in β1 subunit knock down group (18.6 ± 1.6 μM) was 877-fold lower than that in the scramble group (21.2 ± 2.5 nM) (Figure 8G and Table 2). Having shown that the IC_50_ of puerarin on Na_v_1.8 in DRG neurons in paclitaxel rats was 606-fold and 2252-fold lower than that in control rats in the resting and inactivated state, respectively. Interestingly, the IC_50_ of puerarin on Na_v_1.8 in DRG neurons in paclitaxel rats with β1 subunit knock down was only 1.3-fold and 1.6-fold lower than that in control rats in the resting and inactivated state, respectively. In other words, the preferential blockage of Na_v_1.8 in DRG neurons of paclitaxel-induced neuropathic pain rats by puerarin was abolished by knock down of the β1 subunit. This finding suggested that the selective inhibition of Na_v_1.8 in neuropathic pain rats by puerarin may occur via blockage of the β1 subunit. Considering that the IC_50_ of Puerarin on inactivated TTX-S sodium channel is lower than that the dose of Puerarin in reducing neuropathic pain (352.9 ± 39.1 nM < 1 μM) (Figures 1C, 7D). Since Na_v_1.7 carries a large portion of the TTX-S sodium current in sensory neurons, so we investigated the effect of puerarin on Na_v_1.7 channel. We found that puerarin (350 nM) reduced the current of Na_v_1.7 in DRG neurons in paclitaxel-induced neuropathic pain rats, however, knock down β1 subunit could not inhibit the effect of puerarin on Na_v_1.7 (Figure 8E). This finding suggested that the inhibition of Na_v_1.7 by puerarin may not occur via blockage of the β1 subunit.

## Discussion

Our results showed that local application of puerarin dose-dependently attenuated paclitaxel-induced mechanical allodynia and thermal hyperalgesia. The current study showed that the IC_50_ of puerarin for resting and inactivated Na_v_ channels in DRG neurons was 105 and 489 times lower in paclitaxel-induced neuropathic pain rats than in control rats. The use-dependent blocking by puerarin was higher in paclitaxel group than in control group at 10 Hz stimulation. In other words, the blocking effects of puerarin on Na_v_ channels in DRG neurons were stronger in neuropathic pain state than that in normal state. These finding suggested that puerarin will have a better analgesic effect on neuropathic pain in the clinic. The use-dependent blockage of the Na_v_ channel by puerarin may also be important for the treatment of epilepsy and arrhythmias in the clinic, because of the selective blockage of channels that open at high frequency (Mantegazza et al., 2010). The same is true in the case of neuropathic pain induced by nerve injury, as the spontaneous activity in DRG neurons is mediated by the repetitive opening of Na_v_ channels at high frequency in L5 spinal nerve injury rats (Liu et al., 2000). Use-dependent blocking arises from binding of drugs to inactivated channels recruited during repetitive pulses and from dissociation of drugs from inactivated states with a time constant slower than the frequency of the pulses (Leffler et al., 2007). Alternatively, it was suggested that use-dependent blocking results from slow recovery of drug-bound channels due to an interaction between drugs and slow inactivated states (Ong et al., 2000). Indeed, slow inactivation is more pronounced in TTX-R compared with TTX-S channels (Rush et al., 1998; Blair and Bean, 2003; Chevrier et al., 2004). Moreover, slow inactivation in TTX-R channels was suggested to play a major role in controlling adaptation of AP firing in nociceptive sensory neurons (Blair and Bean, 2003). Our results showed that puerarin has a significant delayed recovery effect on sodium channels, which may explain that puerarin has a stronger use-dependent blocking effect on sodium channels. The difference of adaptation during the 20 first pulses between control and paclitaxel group may due to paclitaxel accelerates recovery of inactivated sodium channels. The current data revealed that puerarin prompted a prominent hyperpolarizing shift in the inactivation and a delay in the recovery time at corresponding IC_50_ concentrations but did not affect the activation. This profile is quite consistent with the possibility that puerarin selectively affects the inactivated Na_v_ channels, as the IC_50_ of puerarin for the inactivated state in the paclitaxel model was 44-fold and in controls was 9-fold lower than that at the resting state in each group. Na_v_ channels go through rapid transitions from the resting state to the open, inactivated state and eventually back to the resting state (Aldrich et al., 1983) and Na_v_ channel inactivation consists of two processes: fast inactivation, occurring on a millisecond time scale, and slow inactivation, occurring within seconds to minutes (Niespodziany et al., 2013). Blocking the different states may lead to various pharmacological effects (Hille, 1977). For example, both lidocaine and bupivacaine completely block the resting Na_v_ channels in DRG neurons at high concentrations (Scholz et al., 1998) and therefore are generally used as local anesthetics. Our study showed that the maximum inhibition rates for resting and inactivated Na_v_ channels produced by puerarin were less than 71% in control and paclitaxel rats. This incomplete blockage may be the basis of the minimal side effect of puerarin observed in the clinic.

Previous studies have shown that Na_v_1.8 channels in DRG neurons are critically involved in neuropathic pain (Porreca et al., 1999; Lai et al., 2002; He et al., 2010) and inflammatory pain (Joshi et al., 2006). The most important finding is that the selective block of Na_v_1.8 channels may remits both of these types of pain (Jarvis et al., 2007). Therefore, Na_v_1.8 channels are an important target when investigating new anti-nociceptive substances. It is well established that synthetic μO-conotoxin selectively blocks rat Na_v_1.8 channels and has potent and long-lasting local anesthetic effects (Ekberg et al., 2006). Our studies showed that puerarin potently blocked Na_v_1.8 channels in resting and inactivated states in a neuropathic pain state, suggesting that puerarin may be effective for the relief of severe pain similar to conotoxin. This is a very interesting question that should be explored further in the clinic.

The Na_v_ channel is composed of a combination of pore-forming α subunits and β subunits (β1-4), which are known as auxiliary components that act in a regulatory capacity (Isom et al., 1994). Compelling studies have shown that all four β subunits are expressed in DRG neurons (O’Malley and Isom, 2015) and β1 increases the fraction of α subunits that operate in a fast gating mode, thus accelerating the activation and inactivation kinetics of the channel and modulating the frequency with which neurons fire (Isom et al., 1992). A previous study showed that the upregulation of the β1 subunit was involved in the generation of neuropathic pain (Blackburn-Munro and Fleetwood-Walker, 1999). Interestingly, co-expression of the β1 subunit induced a 2.3-fold increase in Na_v_1.8 current density in HEK293 cells (Zhao et al., 2011). Our study showed that puerarin has a stronger blocking effect on the Na_v_1.8 subtype in a neuropathic pain state than a normal state, and this selective inhibitory effect depends on the β1 subunit. This finding suggests the possibility that the β1 subunit is a potential target of puerarin and reminds us that the treatment of chronic pain should not only focus on α subunit of Na_v_ channel but also pay attention to β1 subunit. Thus, the β1 subunit probably is an emerging therapeutic target (O’Malley and Isom, 2015).

Recent research revealed that puerarin has an anticancer effect on tumor cells. For example, puerarin inhibits non-small cell lung cancer cell growth via the induction of apoptosis (Hu et al., 2018). Puerarin suppress bladder cancer cell proliferation through the mTOR/p70S6K signaling pathway (Jiang et al., 2018). In clinic, paclitaxel is commonly used to treat patients with non-small cell lung cancer (Sandler et al., 2006). Our studies showed that puerarin significantly inhibits chemotherapy paclitaxel-induced neuropathic pain. Therefore, for non-small cell lung cancer patients requiring chemotherapy the use of puerarin may alleviate chemotherapy induced pain as well as reduce cancer cell proliferation. However, more clinical trials are needed in the further study.

As we known that potassium channel plays a critical role in the repolarization of AP. The half-width of AP can be increased by decreasing the function or expression of potassium channel (Busserolles et al., 2016). Our results showed that paclitaxel increased the half-width of AP indicated that paclitaxel may affect potassium channel (Jaggi and Singh, 2012). The duration of the spike was also increased after puerarin treatment. The reason for this phenomenon may be that puerarin acts on potassium channel of DRG neuron. So further research is needed. Previous study demonstrates that puerarin attenuates nerve injury-induced neuropathic pain by modulating P2X3 receptors (Xu et al., 2012) and this is a chronic process (day 14). While our study reveals the acute effect (15 min to 6 h) of puerarin on sodium channels.

In summary, this is the first study to report that puerarin may preferentially block the β1 subunit of Na_v_1.8 and reduced the excitability of DRG neurons contributes to its anti-paclitaxel induced neuropathic pain effect. These findings may provide evidence for a new area of clinical application of puerarin: chemotherapy-induced peripheral neuropathy (CIPN).

## Author Contributions

X-LZ performed the experiments. X-YC prepared the figures. R-CL and W-AZ analyzed and interpreted the data. M-XX edited the manuscript. All authors finally approved the version to be published and agreed to be accountable for all aspects of the work in ensuring that questions related to the accuracy or integrity of any part of the work are appropriately investigated and resolved.

## Conflict of Interest Statement

The authors declare that the research was conducted in the absence of any commercial or financial relationships that could be construed as a potential conflict of interest.
